# Relationship between soil carbon stocks and crop yields in two dryland cropping systems

**DOI:** 10.1002/jeq2.70312

**Published:** 2026-11-10

**Authors:** Upendra M. Sainju, Brett L. Allen, Jalal D. Jabro, William B. Stevens

**Affiliations:** ^1^ USDA‐ARS Northern Plains Agricultural Research Laboratory Sidney Montana USA

## Abstract

The relationships among soil total C (STC), soil inorganic C (SIC), and soil organic C (SOC) stocks and crop yields in dryland cropping systems are not adequately known. We measured STC, SIC, and SOC stocks at the 0‐ to 120‐cm depth and crop yields at two dryland farming sites (Froid and Sidney, MT) after 10–11 years of crop rotations in the United States northern Great Plains. Crop rotations in Froid included rotations of durum (*Triticum turgidum* L.), camelina (*Camelina ceantz* L.), pea (*Pisum sativum* L.), canola (*Brassica napus* L.), and safflower (*Carthamaus tinctorius* L.). Crop rotations in Sidney were rotations of barley (*Hordeum vulgare* L.), pea, camelina, canola, and spring and winter wheat (*Triticum aestivum* L.). Highly diversified crop rotations and monocropping increased SOC and STC compared to less diversified rotations, but SIC varied by crop rotation. The principal component analysis showed that STC and SOC at 0–15, 15–30, 30–60, 60–90, 90–120, and 0–120 cm were related to each other, but the relationship of SIC with STC and SOC varied by site. At Froid, SIC at 0–15 cm was related, but STC and SOC at all depths were not related to crop yield. At Sidney, SIC at 0–15, 90–120, and 0–120 cm was related to crop yield. The SOC at 30–60, 60–90, 0–30, 0–60, 0–90, and 0–120 cm was more closely related to crop yield than STC. The SOC can be used for measuring soil C sequestration and relating to crop yields in dryland cropping systems.

## Plain Language Summary

Soil carbon stocks are important for measuring carbon sequestration, soil health, and environmental quality, but little is known about the relationship between soil carbon stocks and crop yields in dryland cropping systems. Researchers at the Agricultural Research Service, Sidney, MT, examined the relationships between soil carbon stocks and crop yields in long‐term crop rotations studies at two dryland farming sites. They reported that soil total carbon and soil organic carbon were related to each other, but soil inorganic carbon was not related to either of them. Soil inorganic carbon at the surface layer was related to crop yield at the site with coarse soil texture, and both soil organic and inorganic carbon at the surface and subsurface layers were related to crop yield at the site with fine soil texture. Producers can use soil organic carbon to predict crop yields in dryland cropping systems.

**Abbreviations**
BP: barley–pea
DC: durum–camelina
DCP: durum–camelina–pea
DS: durum–safflower
DSP: durum–safflower–pea
DN: durum–canola
DNP: durum–canola–pea
DP: durum–pea
DPC: durum–pea–camelina
DPF: durum–pea–safflower
DPN: durum–pea–canola
PBCS: pea–barley–camelina–spring wheat
PBNS: pea–barley–canola–spring wheat
PC: principal component
PCA: principal component analysis
PWCS: pea–winter wheat–camelina–spring wheat
PWNS: pea–winter wheat–canola–spring wheat
SIC: soil inorganic C
SOC: soil organic C
SP: spring wheat–pea
STC: soil total C
WP: winter wheat–pea

## 1 INTRODUCTION

Soils in dryland cropping systems in arid and semiarid regions are enriched with soil inorganic C (SIC), especially at subsurface layers (Lal, 2004). This occurs because of the formation of secondary carbonates from the dissolution of CO_2_ and eventual precipitation of CaCO_3_ and MgCO_3_ in calcic and petrocalcic horizons (Cihacek & Ulmer, 2002; Lal, 2004; Monger et al., 1991). Carbon sequestration due to agricultural management practices and variations in climatic conditions occurs both in SIC and soil organic C (SOC) in dryland soils (Cihacek & Ulmer, 2002; Lal, 2004; Monger et al., 1991). Because of the occupation of large area, dryland soils are one of largest pools of global C stocks and C cycling in the terrestrial ecosystem (Eswaran et al., 2000; Lal, 2004; Mikhailova & Post, 2006; Sahrawat, 2003). Carbon sequestration in soils, however, is mostly measured by changes in SOC over time (Food and Agricultural Organization [FAO], 2004; United States Environmental Protection Agency [USEPA], 2011), but sequestration due to changes in SIC stocks is largely unknown (Cihacek & Ulmer, 2002; Lal, 2004; Monger et al., 1991). Enrichment of SOC and SIC due to novel management strategies, such as cropping systems and crop rotations, can enhance soil health by improving soil physical, chemical, and microbial properties and environmental quality by reducing CO_2_ emissions, a major greenhouse gas that contributes to global warming (Food and Agricultural Organization [FAO], 2004; United States Environmental Protection Agency [USEPA], 2011).

The measurement of SOC in dryland soils involves determinations of soil total C (STC) and SIC, as SOC is calculated by deducting SIC from STC. This results in additional expenses of measuring SOC that includes two‐step processes of measuring STC and SIC compared to STC, which involves only a one‐step process. Sainju et al. (2021, 2022) reported that STC at 0–15 cm was related to greater number of soil physical, chemical, and microbial properties and had a stronger relationship with crop yields than SOC in dryland cropping systems due to inclusion of SIC with SOC. If SOC can be replaced by STC for measuring soil C sequestration that affects soil health and environmental quality, it can save labor, cost, and time for measuring C stocks in dryland cropping systems.

Crop rotations can affect STC, SIC, and SOC variably due to differences in the quality and quantity of crop residue returned to the soil. While several researchers (Asraf et al., 2025; Sainju, 2014) found that legume–nonlegume crop rotations increased SOC compared to continuous nonlegumes due to greater crop residue returned to the soil, others (King & Blesh, 2018; Zhang et al., 2025) reported the opposite effect due to enhanced mineralization of legume residues stemming from lower C/N ratios. Diversified crop rotations that include legumes, nonlegumes, and oilseed crops with greater rotation lengths and continuous nonlegumes that return higher amounts of crop residue to the soil can enhance SOC compared to less diversified rotations (Alhameid et al., 2017; Fu et al., 2017; Jarecki et al., 2018; Lehman et al., 2017; Schmer et al., 2020). Crop rotations and continuous nonlegumes with higher crop residue C/N ratios can enhance STC compared to rotations with lower residue C/N ratios (Sainju et al., 2025). Sainju, Lenssen, et al. (2020) reported that crop rotations had little effect on STC and SIC after 4 years. These studies indicate that crop species, crop rotation length, and duration of the study have substantial impact on SOC, STC, and SIC.

The STC, SIC, and SOC stocks have variable relationships among themselves and crop yields. Sainju et al. (2021) showed that SOC at 0–15 cm was related to STC and SIC and that STC was more related to crop yield than SOC, but SIC was not related to crop yield. Numerous researchers (Bauer & Black, 1994; Culman et al., 2012, 2013; Hurisso et al., 2016; Lal, 2020) also showed a positive relationship between SOC and crop yield, as SOC helps to enhance crop yield by conserving soil water during drought (Diaz‐Zorita et al., 1999; Lal, 2020). Increased SOC also enhances microbial activity that increases nutrient mineralization and availability, thereby favoring crop yields (Culman et al., 2013; Dick & Gregorich, 2004).

The relationships between soil C stocks and crop yields have been limited only to a depth of 15 cm of the soil profile (Culman et al., 2012, 2013; Hurisso et al., 2016; Sainju et al., 2021, 2022). In dryland cropping systems, SIC increases with depth and occurs more in the subsurface than surface soil layers (Lal, 2004; Sainju, Lenssen, et al., 2020). Also, crop roots grow to a depth of >1 m for water absorption in dryland soils, which can provide significant C input for enhancing soil C stock (Bollinder et al., 1997; Caires et al., 2008; Gan et al., 2009; Liu et al., 2011). Therefore, it is imperative that relationships between soil C stocks and crop yields be examined at depth layers >15 cm. Although STC may not be an independent variable to relate with SOC, SIC, and crop yield because it is calculated as the sum of SOC and SIC, the increased cost, labor, and time of analyzing SOC and better relationship of STC than SOC at 0–15 cm with crop yields in dryland cropping systems (Sainju et al., 2021, 2022) suggest that further exploration of the relationship between STC, SOC, and SIC at deep soil layers and crop yield is needed. We evaluated relationships among STC, SIC, and SOC stocks at 0‐ to 15‐cm, 15‐ to 30‐cm, 30‐ to 60‐cm, 60‐ to 90‐cm, 90‐ to 120‐cm, 0‐ to 30‐cm, 0‐ to 60‐cm, 0‐ to 90‐cm, and 0‐ to 120‐cm depths and crop yields at two dryland farming sites managed with 10–11 years of diversified crop rotations in the United States northern Great Plains. Our research questions were as follows: (1) Are STC, SIC, and SOC at various depth layers related to each other? (2) Do soil C stocks relate to crop yields? We hypothesized that STC and SOC would be closely related to each other as well as to crop yields, but SIC would not be related to these parameters. Our objectives were to (1) determine the effect of crop rotations on STC, SIC, and SOC stocks at 0–120 cm and crop yields and (2) relate soil C stocks and crop yields at two dryland farming sites planted with 10–11 years of diversified crop rotations in the United States northern Great Plains.

## 2 MATERIALS AND METHODS

### 2.1 Field experiments

The field experiments in dryland farming sites have been described in detail by Sainju, Allen, et al. (2020), Sainju et al. (2025). Briefly, the studies were conducted from 2012 to 2021 at Froid and from 2012 to 2022 at Sidney, MT. The Froid site (48° 15′ N, 104° 30′ W) had a mean annual air temperature of 7.0°C, annual precipitation of 341 mm, and Dooley sandy loam (fine loamy, mixed, frigid, Typic Argiboroll) soil with 645 g kg^−1^ sand, 185 g kg^−1^ silt, 170 g kg^−1^ clay, and 7.2 pH at the 0‐ to 15‐cm depth. The Sidney site (47° 46′ N, 104° 15′ W) had mean annual air temperature of 7.4°C, annual precipitation of 357 mm, and Williams loam (fine‐loamy, mixed, superactive, frigid, Typic Argiustolls) soil with 350 g kg^−1^ sand, 325 g kg^−1^ silt, 325 g kg^−1^ clay, and 6.1 pH at the 0‐ to 15‐cm depth. The STC, SIC, and SOC stocks at 0–15, 15–30, 30–60, 60–90, 90–120, and 0–120 cm at the beginning of the experiment in 2012 at Froid and Sidney are shown in Table 1. The sites had been previously cropped (10 years) with spring wheat–fallow and spring wheat–safflower–fallow rotations.

**TABLE 1:** Soil total C (STC), soil inorganic C (SIC), and soil organic C (SOC) stocks at the beginning of the experiment in 2012 at Froid and Sidney.

Soil depth	Froid			Sidney		
	STC (Mg C ha^−1^)	SIC (Mg C ha^−1^)	SOC (Mg C ha^−1^)	STC (Mg C ha^−1^)	SIC (Mg C ha^−1^)	SOC (Mg C ha^−1^)
0–15 cm	13.14	0.0	13.14	34.1	0.0	34.1
15–30 cm	12.90	3.6	9.26	20.2	0.0	20.2
30–60 cm	92.28	7.6	84.73	113.0	72.0	41.0
60–90 cm	108.38	88.0	20.38	158.6	72.4	86.2
90–120 cm	116.18	65.1	51.10	149.3	58.1	91.2
0–120 cm	342.88	164.27	178.61	475.25	202.55	272.70

Crop rotations at Froid were continuous durum (D), durum–camelina (DC), durum–camelina–pea (DCP), durum–canola (DN), durum–canola–pea (DNP), durum–pea (DP), durum–pea–camelina (DPC), durum–safflower (DF), durum–pea–safflower (DPF), durum–safflower–pea (DFP), and durum–pea–canola (DPN). At Sidney, crop rotations were continuous barley (B), barley–pea (BP), pea–barley–camelina–spring wheat (PBCS), pea–barley–canola–spring wheat (PBNS), pea–winter wheat–camelina–spring wheat (PWCS), pea–winter wheat–canola–spring wheat (PWNS), continuous spring wheat (S), spring wheat–pea (SP), continuous winter wheat (W), and winter wheat–pea (WP). All phases of the crop rotations were present each year. An example of sequence of crops in crop rotations when all phases of crops are present every year is shown by Sainju, Lenssen et al. (2020). Crop rotations were arranged in a randomized block design with four replications. The plot size was 12.2 m × 15.2 m at Froid and 12.2 m ×12.2 m at Sidney. Both experiments were conducted in the no‐till conditions.

Crops were seeded from late April to early May at a row spacing of 20 cm with a no‐till drill each year, except for winter wheat, which was seeded in September of the previous year. Seeding rates for spring and winter wheat were 2.2 million seeds ha^−1^, durum 3.0 million seeds ha^−1^, barley 1.1 million seeds ha^−1^, pea 0.75 million seeds ha^−1^, canola 7 kg ha^−1^, camelina 9 kg ha^−1^, and safflower 28 kg ha^−1^. Nitrogen fertilizers as urea and monoammonium phosphate were banded, 5 cm to the side and 5 cm below the seed, at 148, 140, 130, 54, and 90 kg N ha^−1^ for spring wheat, winter wheat, durum, barley, and safflower, respectively, as a basic dose at planting. For canola and camelina, N fertilizers as urea, monoammonium phosphate, and ammonium sulfate were applied at 117 and 90 kg N ha^−1^, respectively, and S fertilizer as ammonium sulfate was applied at 27 kg S ha^−1^ for both crops at planting. Pea received N fertilizer from monoammonium phosphate at 5 kg N ha^−1^. Because of the presence of soil residual NO_3−_‐N that negatively affects soil and environmental quality, N fertilization rates for all crops, except pea, were adjusted to soil residual NO_3_‐N content to a depth of 60 cm determined in the autumn of the previous year. Therefore, desired N fertilization rates included both soil and fertilizer N. At planting, P fertilizer at 13 kg P ha^−1^ as monoammonium phosphate and K fertilizer at 22 kg K ha^−1^ as muriate of potash were banded to all crops, 5 cm to the side and 5 cm below the seed. Herbicides and pesticides were applied to crops as needed to control weeds and pests. In August–September, grains were harvested using a combine harvester and crop residues were returned to the soil. Grain yield was determined in an oven‐dried basis by weighing the harvested grains after oven drying a sample at 70°C for 7 days. Annualized crop yield for a rotation was determined by averaging yields of all crops within the rotation in a year (Sainju, Lenssen, et al., 2020).

### 2.2 Soil sampling and analysis

Soil samples were collected at 0–120 cm from four locations within a plot to reduce spatial heterogeneity using a hydraulic probe (3.5 cm inside diameter) mounted on a truck in October 2021 at Froid and in October 2022 at Sidney. Soil cores were divided into 0‐ to 15‐cm, 15‐ to 30‐cm, 30‐ to 60‐cm, 60‐ to 90‐cm, and 90‐ to 120‐cm depth intervals and composited by depth. Soil samples were air‐dried and ground to 2 mm. Because of the soil disturbance, these cores could not be used for measuring soil bulk density. Therefore, for determination of the soil bulk density, a separate undisturbed core was collected from each plot at the same depth intervals as above and oven dried at 110°C for 24 h. The bulk density was determined by dividing the weight of the oven‐dried soil by the volume of the core. The SIC concentration was determined using a calcimeter (Lita Analytical) and the STC concentration using a C and N analyzer (Elementar) after grinding a subsample of soil to 0.5 mm. The SIC and STC stocks were calculated by multiplying SIC and STC concentrations by the bulk density and the thickness of the soil layer. The SOC stock was calculated by deducting the SIC stock from the STC stock. The STC, SIC, and SOC stocks at 0–30, 0–60, 0–90, and 0–120 cm were calculated by adding stocks from individual depth layers.

### 2.3 Relationship between soil carbon stocks and crop yields

It does not make sense to relate soil C stocks with yields of individual crops in the crop rotation because the rotation contains a combination of crops. Therefore, we used the annualized crop yield of a rotation rather than individual crop yield to relate with soil C stocks as stated by various researchers (Culman et al., 2013; Lucas & Weil, 2012; Sainju et al., 2021, 2022; van Es & Karlen, 2019). Use of annualized price of crops as a metric instead of annualized crop yield in a crop rotation to relate with soil C stocks adds another uncertainty because the price changes every year that can jeopardize the relationship. Because continuous return of crop residue to the soil over years can increase C sequestration, soil health, and fertility (Culman et al., 2013; Lucas & Weil, 2012; van Es & Karlen, 2019), we used data for soil C stocks and crop yields at the end of the two experiments to relate each other to determine if increased soil C stocks can enhance crop yields through improved soil water, nutrients, and plant growth relationships.

### 2.4 Data analysis

Data for crop yields and STC, SIC, and SOC stocks at a depth interval were analyzed by site using a SAS‐MIXED procedure, SAS/STAT version 6.9 (Littell et al., 2006). Data were subjected to normality and homogeneity of variance tests (Littell et al., 1991) before being analyzed by the SAS‐MIXED procedure. Crop rotation was considered as the fixed effect and replication as the random effect for data analysis. Means were separated using the least square means test (Littell et al., 2006) when significant. For relating soil C stocks and crop yields, data were analyzed using the principal component analysis (PCA) with the PROC FACTOR procedure of SAS (Littell et al., 2006). Parameters are positively and closely related to each other when the principal component (PC) vectors are at an acute angle to each other, not related when the vectors are at a right angle, and negatively related when the vectors are at an obtuse angle. Pearson's correlation analysis was used to determine correlations among soil C stocks and crop yields. Statistical significance was observed at *p* ≤ 0.05, unless otherwise noted.

## 3 RESULTS

### 3.1 Soil bulk density

Soil bulk density was unaffected by crop rotation both at Froid and Sidney (Table 2). Bulk density increased with depth at both sites, probably due to the greater presence of carbonate nodules in calcic and petrocalcic layers. Bulk density at 0–15 cm ranged from 1.36 Mg m^−3^ for DP to 1.43 Mg m^−3^ for DFP at Froid. At 90–120 cm, bulk density ranged from 1.49 Mg m^−3^ for DFP to 1.69 Mg m^−3^ for DP. Similarly, bulk density at 0–15 cm ranged from 1.32 Mg m^−3^ for PWCS to 1.41 Mg m^−3^ for W and WP at Sidney. At 90–120 cm, bulk density ranged from 1.57 Mg m^−3^ for PBCS to 1.71 Mg m^−3^ for PWNS. Changes in bulk density due to crop rotation may affect soil C stocks, as described below.

**TABLE 2:** Effect of crop rotation on soil bulk density at the 0‐ to 120‐cm depth at Froid and Sidney.

Crop rotation^a^	Soil depth				
	0–15 cm	15–30 cm	30–60 cm	60–90 cm	90–120 cm
	Bulk density (Mg m^−3^)				
	**Froid**				
D	1.37	1.42	1.45	1.58	1.63
DC	1.40	1.45	1.42	1.60	1.60
DCP	1.41	1.49	1.45	1.60	1.64
DN	1.37	1.49	1.43	1.55	1.57
DNP	1.38	1.52	1.44	1.54	1.62
DP	1.36	1.47	1.42	1.62	1.69
DPC	1.40	1.46	1.49	1.57	1.61
DPN	1.40	1.49	1.46	1.59	1.62
DPF	1.38	1.45	1.42	1.57	1.64
DfF	1.41	1.51	1.45	1.61	1.61
DFP	1.43	1.47	1.47	1.57	1.49
*p* value	0.756	0.320	0.804	0.938	0.134
	**Sidney**				
B	1.39	1.32	1.41	1.58	1.70
BP	1.39	1.31	1.36	1.48	1.65
PBCS	1.37	1.32	1.37	1.46	1.57
PBNS	1.36	1.29	1.35	1.48	1.59
PWCS	1.38	1.27	1.36	1.51	1.63
PWNS	1.32	1.29	1.37	1.52	1.71
S	1.43	1.23	1.37	1.47	1.60
SP	1.33	1.27	1.32	1.48	1.62
W	1.41	1.30	1.34	1.49	1.59
WP	1.41	1.26	1.35	1.50	1.59
*p* value	0.223	0.382	0.585	0.511	0.701

Crop rotation in Sidney are: BP, barley–pea; PBCS, pea–barley–camelina–spring wheat; PBNS, pea–barley–canola–spring wheat; PWCS, pea–winter wheat–camelina–spring wheat; PWNS, pea–winter wheat–canola–spring wheat; SP, spring wheat–pea; WP, winter wheat–pea.

^a^ Crop rotations in Froid are D, continuous durum; DC, durum–camelina; DCP, durum–camelina–pea; DN, durum–canola; DNP, durum–canola–pea; DP, durum–pea; DPC, durum–pea–camelina; DPN, durum–pea–canola; DPF, durum–pea–safflower; DF, durum–safflower; and DFP, durum–safflower–pea.

### 3.2 Soil carbon stocks

Crop rotation affected STC and SOC at 0–15 cm at Froid (Table 3). At Sidney, crop rotation affected STC at 15–30 cm, SIC at 90–120 and 0–120 cm, and SOC at 15–30, 30–60, 90–120, and 0–120 cm. At Froid, STC and SOC at 0–15 cm were 15%–19% greater for D than DNP, DP, DPC, DPN, and DPF (Table 4). The STC and SOC were not significantly different among crop rotations at other depths. There was also no significant difference in SIC among crop rotations at all depths. At Sidney, STC at 15–30 cm was 14%–55% greater for B and PWCS than BP, PBCS, PBNS, PWNS, S, SP, W, and WP (Table 5). At other depths, STC did not significantly differ among crop rotations. The SIC at 90–120 cm was 17%–39% greater for BP than other crop rotations, except B and S. At 0–120 cm, SIC was 11%–22% greater for B than other crop rotations, except BP and PWNS. The SOC at 15–30 cm was 43%–84% greater for B, PBNS, PWCS, PWNS, and S than BP, SP, and W. At 30–60 cm, SOC was 58%–74% greater for PBCS than BP and SP. At 90–120 cm, SOC was 36%–48% greater for PWNS and W than BP, PBNS, S, and WP. At 0–120 cm, SOC was 31%–46% greater for B, PBCS, PWCS, PWNS, and W than BP.

**TABLE 3:** Analysis of variance for the effect of crop rotation on soil total C (STC), soil inorganic C (SIC), and soil organic C (SOC) at Froid and Sidney.

Significance	Soil depth					
	0–15 cm	15–30 cm	30–60 cm	60–90 cm	90–120 cm	0–120 cm
	**Froid**					
	STC					
*df*	10	10	10	10	10	10
*F* value	2.67	0.53	1.04	1.11	0.44	1.11
*p* value	**0.03**	0.86	0.42	0.36	0.92	0.36
	SIC					
*df*	10	10	10	10	10	10
*F* value	0.75	0.84	1.31	0.83	1.17	1.34
*p* value	0.67	0.60	0.24	0.60	0.32	0.22
	SOC					
*df*	10	10	10	10	10	10
*F* value	2.56	0.82	0.82	0.83	0.45	0.98
*p* value	**0.03**	0.61	0.61	0.60	0.92	0.46
	**Sidney**					
	STC					
*df*	9	9	9	9	9	9
*F* value	0.72	2.85	1.11	0.91	1.17	1.35
*p* value	0.69	**0.02**	0.36	0.52	0.32	0.23
	SIC					
*df*	9	9	9	9	9	9
*F* value	0.59	0.56	0.90	1.33	1.93	2.63
*p* value	0.80	0.83	0.53	0.23	**0.05**	**0.02**
	SOC					
*df*	9	9	9	9	9	9
*F* value	0.41	2.22	2.36	1.62	2.01	2.69
*p* value	0.93	**0.04**	**0.03**	0.12	**0.05**	**0.01**

*Note*: Bold numbers denote significant *p* values.

**TABLE 4:** Effect of crop rotation on soil total C (STC), soil inorganic C (SIC), and soil organic C (SOC) stocks at the 0‐ to 120‐cm depth at Froid.

Crop rotation^a^	Soil depth					
	0–15 cm	15–30 cm	30–60 cm	60–90 cm	90–120 cm	0–120 cm
	**STC (Mg C ha^−1^)**					
D	20.6a	14.2	49.1	86.2	107.9	278.1
DC	19.3ab	16.2	84.0	122.7	112.8	355.2
DCP	18.8ab	20.6	75.1	101.6	104.8	320.9
DN	19.5ab	14.1	56.3	107.8	110.0	307.1
DNP	17.8b	15.9	65.8	113.3	103.7	316.5
DP	17.9b	16.7	65.9	115.0	107.2	322.6
DPC	17.8b	15.8	95.7	113.7	111.0	353.9
DPN	17.3b	15.8	89.3	122.7	109.2	354.2
DPF	17.6b	14.9	69.2	111.9	98.8	312.4
DF	19.0ab	16.0	81.8	112.8	106.2	335.7
DFP	18.3ab	18.2	78.2	109.4	100.5	324.5
	**SIC (Mg C ha^−1^)**					
D	0.0	1.7	13.7	73.7	70.1	159.1
DC	0.1	1.9	22.1	83.7	67.9	175.4
DCP	0.1	2.3	28.3	81.0	60.5	172.1
DN	0.0	1.9	20.0	88.2	71.0	181.2
DNP	0.1	2.8	21.8	81.0	66.9	172.5
DP	0.1	1.9	20.0	80.5	63.6	165.9
DPC	0.1	2.1	25.9	84.8	63.5	176.4
DPN	0.1	3.4	37.7	86.6	72.3	200.1
DPF	0.0	3.5	24.5	89.2	66.4	183.7
DF	0.1	4.5	32.8	91.7	66.3	195.4
DFP	0.4	3.1	26.4	82.9	61.0	173.7
	**SOC (Mg C ha^−1^)**					
D	20.6a	12.5	35.4	12.5	37.9	118.9
DC	19.2ab	14.3	61.7	36.9	44.7	179.2
DCP	18.8ab	18.2	46.9	20.5	44.4	148.8
DN	19.5ab	12.1	36.3	19.5	39.1	126.5
DNP	17.8b	13.1	44.0	32.3	36.8	144.1
DP	17.9b	14.8	45.9	34.5	43.6	156.8
DPC	17.8b	13.7	69.8	28.9	47.4	177.6
DPN	17.3b	12.3	51.6	36.1	36.9	154.2
DPF	17.5b	11.3	44.7	22.7	32.5	128.8
DF	19.0ab	11.5	49.4	21.3	40.3	141.1
DFP	17.9b	15.1	51.8	26.6	39.6	150.9

*Note*: Numbers following different letters within a column in a set are significantly different at *p* ≤ 0.05 by the least square means test.

^a^ Crop rotations are D, continuous durum; DC, durum–camelina; DCP, durum–camelina–pea; DN, durum–canola; DNP, durum–canola–pea; DP, durum–pea; DPC, durum–pea–camelina; DPN, durum–pea–canola; DPF, durum–pea–safflower; DF, durum–safflower; and DFP, durum–safflower–pea.

**TABLE 5:** Effect of crop rotation on soil total C (STC), soil inorganic C (SIC), and soil organic C (SOC) stocks at the 0‐ to 120‐cm depth at Sidney.

Crop rotation^a^	Soil depth					
	0–15 cm	15–30 cm	30–60 cm	60–90 cm	90–120 cm	0–120 cm
	**STC (Mg C ha^−1^)**					
B	24.4	47.1a	151.2	143.9	136.7	503.4
BP	25.7	30.4c	128.2	131.8	139.2	455.2
PBCS	25.0	36.6bc	146.4	136.4	128.7	473.1
PBNS	24.5	40.0b	131.5	128.2	121.9	446.0
PWCS	26.5	45.7a	134.9	136.0	126.9	469.9
PWNS	26.1	37.6b	136.8	136.8	141.1	478.3
S	25.2	37.7b	145.8	145.3	122.0	476.0
SP	24.4	31.7c	124.1	133.9	125.0	439.1
W	28.4	36.1bc	128.7	139.3	133.6	166.1
WP	24.8	32.8bc	135.2	134.7	124.1	451.6
	**SIC (Mg C ha^−1^)**					
B	0.0	18.6	115.9	106.3	89.6ab	330.5a
BP	0.0	15.2	100.0	109.5	98.5a	323.2ab
PBCS	0.0	11.1	97.4	94.4	70.9c	279.8c
PBNS	0.0	12.0	104.6	97.6	76.9bc	295.2bc
PWCS	1.6	17.1	96.7	101.2	81.0bc	298.6bc
PWNS	0.0	10.9	99.3	101.2	82.2bc	293.6bc
S	0.0	10.3	111.9	110.9	84.8ab	317.9ab
SP	0.5	11.8	93.0	91.7	73.5c	270.5c
W	0.0	18.2	102.2	92.3	78.0bc	290.8bc
WP	0.1	11.8	96.5	101.0	84.3b	293.8bc
	**SOC (Mg C ha^−1^)**					
B	24.4	28.4a	35.3ab	37.6	47.1ab	172.9a
BP	25.6	15.2b	28.2b	22.3	40.7b	132.0b
PBCS	25.0	25.5ab	49.0a	41.9	51.8ab	193.3a
PBNS	24.5	28.0a	26.9ab	30.6	40.9b	150.8ab
PWCS	24.9	28.6a	38.2ab	34.8	44.9ab	171.3a
PWNS	26.1	26.7a	37.5ab	35.5	58.9a	184.7a
S	25.1	27.4a	34.0ab	34.4	37.2b	158.1ab
SP	23.8	19.8b	31.1b	42.3	51.5ab	168.6ab
W	28.4	17.9b	26.5ab	47.0	55.6a	175.4a
WP	24.8	21.0ab	38.6ab	33.6	39.8b	157.9ab

*Note*: Numbers following different letters within a column in a set are significantly different at *p* ≤ 0.05 by the least square means test.

^a^ Crop rotations are B, continuous barley; BP, barley–pea; PBCS, pea–barley–camelina–spring wheat; PBNS, pea–barley–canola–spring wheat; PWCS, pea–winter wheat–camelina–spring wheat; PWNS, pea–winter wheat–canola–spring wheat; S, continuous spring wheat; SP, spring wheat–pea; W, continuous winter wheat; and WP, winter wheat–pea.

### 3.3 Crop yields

Crop rotation affected crop yield both at Froid and Sidney (Table 6). Annualized crop yield was 23%–48% greater for DCP than D, DC, DN, DPC, DPN, DPF, DF, and DFP at Froid. At Sidney, annualized crop yield was 41%–192% greater for BP than B, PBCS, PBNS, PWCS, PWNS, S, and WP. Annualized crop yield was also 84%–163% greater for PBCS, PBNS, PWCS, PWNS, SP, W, and WP than S.

**TABLE 6:** Effect of crop rotation on annualized crop yield (2021 at Froid and 2022 at Sidney) and mean crop residue yield across years at Froid and Sidney.

Froid			Sidney		
Crop rotation^a^	Crop yield (Mg ha^−1^)	Crop residue yield (Mg ha^−1^)	Crop rotation^a^	Crop yield (Mg ha^−1^)	Crop residue yield (Mg ha^−1^)
D	0.89b	1.42a	B	0.55bc	3.41c
DC	0.94b	1.00d	BP	1.11a	3.94b
DCP	1.18a	1.35ab	PBCS	0.77b	4.04b
DN	0.80b	1.32b	PBNS	0.70b	4.28a
DNP	0.95b	1.36ab	PWCS	0.75b	4.27a
DP	1.00ab	1.26c	PWNS	0.77b	4.30a
DPC	0.87b	1.12d	S	0.38c	3.99b
DPN	0.89b	1.37ab	SP	0.91ab	4.08b
DPF	0.83b	1.41a	W	1.00ab	3.96b
DF	0.90b	1.24c	WP	0.79b	3.94b
DFP	0.96b	1.35ab			
Significance					
*df*	10	10		9	9
F value	2.23	9.13		2.03	2.47
*p* value	**0.02**	**<0.001**		**0.03**	**0.009**

*Note*: Numbers following different letters within a column are significantly different at *p* ≤ 0.05 by the least square means test. Bold numbers denote significant *p* values.

^a^ Crop rotations at Froid are D, continuous durum; DC, durum–camelina; DCP, durum–camelina–pea; DN, durum–canola; DNP, durum–canola–pea; DP, durum–pea; DPC, durum–pea–camelina; DPN, durum–pea–canola; DPF, durum–pea–safflower; DF, durum–safflower; and DFP, durum–safflower–pea. At Sidney, crop rotations are B, continuous barley; BP, barley–pea; PBCS, pea–barley–camelina–spring wheat; PBNS, pea–barley–canola–spring wheat; PWCS, pea–winter wheat–camelina–spring wheat; PWNS, pea–winter wheat–canola–spring wheat; S, continuous spring wheat; SP, spring wheat–pea; W, continuous winter wheat; and WP, winter wheat–pea.

### 3.4 Correlations among soil carbon stocks and crop yields

At Froid, STC was positively and strongly correlated with SOC at all depths (Table 7). The STC was positively and weakly to strongly correlated with SIC at all depths, except at 0–15 and 60–90 cm. The SOC was negatively and weakly to strongly correlated with SIC at 0–15, 60–90, and 0–120 cm, but correlated positively and moderately with SIC at 30–60 and 0–30 cm. The STC at 30–60, 0–60, and 0–120 cm was negatively and weakly correlated with crop yield. The SIC at 0–15 cm was positively and moderately correlated with crop yield, but at 30–60, 60–90, 90–120, 0–60, 0–90, and 0–120 cm was negatively and weakly to moderately correlated with crop yield. The SOC at 15–30 and 0–30 cm was positively and weakly correlated with crop yield, but at 30–60 cm was negatively and weakly correlated with crop yield.

**TABLE 7:** Correlations among soil total C (STC), soil inorganic C (SIC), soil organic C (SOC) stocks at the 0‐ to 120‐cm depth and crop yield at Froid (*n* = 108).

Parameter	STC	SIC	SOC	Crop yield
	**0–15 cm**			
STC	–	−0.05	0.98***	0.01
SIC	−0.05	–	−0.23*	0.34**
SOC	0.98***	−0.23*	–	−0.01
Crop yield	0.01	0.34**	−0.01	–
	**15–30 cm**			
STC	–	0.41***	0.93***	0.14
SIC	0.41***	–	0.06	−0.09
SOC	0.93***	0.06	–	0.19*
Crop yield	0.14	−0.09	0.19*	–
	**30–60 cm**			
STC	–	0.58***	0.90***	−0.26**
SIC	0.58***	–	0.36**	−0.23*
SOC	0.90***	0.36**	–	−0.24*
Crop yield	−0.26**	−0.23*	−0.24*	–
	**60–90 cm**			
STC	–	0.11	0.80***	−0.09
SIC	0.11	–	−0.42***	−0.21*
SOC	0.80***	−0.42***	–	0.05
Crop yield	−0.09	−0.21*	0.05	–
	**90–120 cm**			
STC	–	0.44***	0.76***	−0.10
SIC	0.44***	–	0.06	−0.28**
SOC	0.76***	0.06	–	0.10
Crop yield	−0.10	−0.28**	0.10	–
	**0–30 cm**			
STC	–	0.37***	0.94***	0.14
SIC	0.37***	–	0.23*	−0.08
SOC	0.94***	0.23*	–	0.19*
Crop yield	0.14	−0.08	0.19*	——
	**0–60 cm**			
STC	–	0.57***	0.90***	−0.21*
SIC	0.57***	–	0.15	−0.34**
SOC	0.90***	0.15	–	−0.18
Crop yield	−0.21*	−0.34**	−0.18	–
	**0–90 cm**			
STC	–	0.44***	0.86***	−0.15
SIC	0.44***	–	−0.06	−0.21*
SOC	0.86***	−0.06	–	−0.10
Crop yield	−0.15	−0.21*	−0.10	–
	**0–120 cm**			
STC	–	0.40***	0.82***	−0.22*
SIC	0.40***	–	−0.19*	−0.29**
SOC	0.82***	−0.19*	–	−0.06
Crop yield	−0.22*	−0.29**	−0.06	–

*, **, and *** denote significance at *p* ≤ 0.05, *p* ≤ 0.01, and *p* ≤ 0.001, respectively.

At Sidney, STC was also positively and strongly correlated with SOC at all depths (Table 8). The STC was positively and strongly correlated with SIC at all depths, except at 0–15 cm. The SOC was negatively and strongly correlated with SIC at 0–15 and 60–90 cm. The STC at 60–90, 90–120, and 0–120 cm was positively and strongly correlated with crop yield. The SIC at 0–15, 90–120, and 0–120 cm was positively and moderately to strongly correlated with crop yield, but at 0–30, 0–60, and 0–90 cm was negatively and weakly correlated with crop yield. The SOC at 30–60, 60–90, 90–120, 0–30, 0–60, and 0–90 cm was positively and moderately to strongly correlated to crop yield, but at 15–30 cm was negatively and weakly correlated with crop yield.

**TABLE 8:** Correlations among soil total C (STC), soil inorganic C (SIC), soil organic C (SOC) stocks at the 0‐ to 120‐cm depth and crop yield at Sidney (*n* = 100).

Parameter	STC	SIC	SOC	Crop yield
	**0–15 cm**			
STC	–	−0.05	0.85***	0.07
SIC	−0.05	–	−0.57***	0.28**
SOC	0.85***	−0.57***	–	−0.04
Crop yield	0.07	0.28**	−0.04	–
	**15–30 cm**			
STC	–	0.65***	0.72***	−0.06
SIC	0.65***	–	−0.06	0.12
SOC	0.72***	−0.06	–	−0.20*
Crop yield	−0.06	0.12	−0.20*	–
	**30–60 cm**			
STC	–	0.57***	0.66***	−0.01
SIC	0.57***	–	0.03	−0.09
SOC	0.66***	0.03	–	0.36**
Crop yield	−0.01	−0.09	0.36**	–
	**60–90 cm**			
STC	–	0.43***	0.45***	0.50***
SIC	0.43***	–	−0.61***	−0.09
SOC	0.45***	−0.61***	–	0.78***
Crop yield	0.50***	−0.09	0.78***	–
	**90–120 cm**			
STC	–	0.57***	0.71***	0.73***
SIC	0.57***	–	−0.17	0.48**
SOC	0.71***	−0.17	–	0.48***
Crop yield	0.73***	0.48**	0.48***	–
	**0–30 cm**			
STC	–	0.64***	0.73***	−0.04
SIC	0.64***	–	−0.06	−0.25*
SOC	0.73***	−0.06	–	0.41***
Crop yield	−0.04	−0.25*	0.41***	–
	**0–60 cm**			
STC	–	0.72***	0.66***	−0.03
SIC	0.72***	–	−0.04	−0.23**
SOC	0.66***	−0.04	–	−0.51***
Crop yield	−0.03	−0.23**	−0.51***	–
	**0–90 cm**			
STC	–	0.60***	0.56***	0.01
SIC	0.60***	–	−0.03	−0.27*
SOC	0.56***	−0.03	–	0.58***
Crop yield	0.01	−0.27*	0.58***	–
	**0–120 cm**			
STC	–	0.53***	0.56***	0.47***
SIC	0.53***	–	−0.09	0.93***
SOC	0.56***	−0.09	–	0.10
Crop yield	0.47***	0.93***	0.10	–

*, **, and *** denote significance at *p* ≤ 0.05, *p* ≤ 0.01, and *p* ≤ 0.001, respectively.

### 3.5 Relationships among soil carbon stocks and crop yields

At Froid, the PC analysis showed that soil C stocks contributed from 45% of the total variability at 90–120 cm to 57% at 30–60 cm for PC1 for the relationship between soil C stock and crop yield (Figures 1 and 3). For PC2, C stocks contributed from 23% of the total variability at 30–60 and 0–60 cm to 36% at 90–120 cm. The largest contributions of the variance in PC1 were SOC and STC at all depths and in PC2 was crop yield. The STC and SOC were closely and positively related to each other at all depths, but SIC was not related to any of these parameters at all depths, except at 30–60 cm. Similarly, STC and SOC at all depths did not relate to crop yield, except at 30–60 cm where a negative relationship occurred between these parameters and at 15–30 and 0–30 cm where a weak positive relationship occurred between SOC and crop yield. The SIC was positively related to crop yield at 0–15 cm but was negatively related at other depths.

**FIGURE 1:**
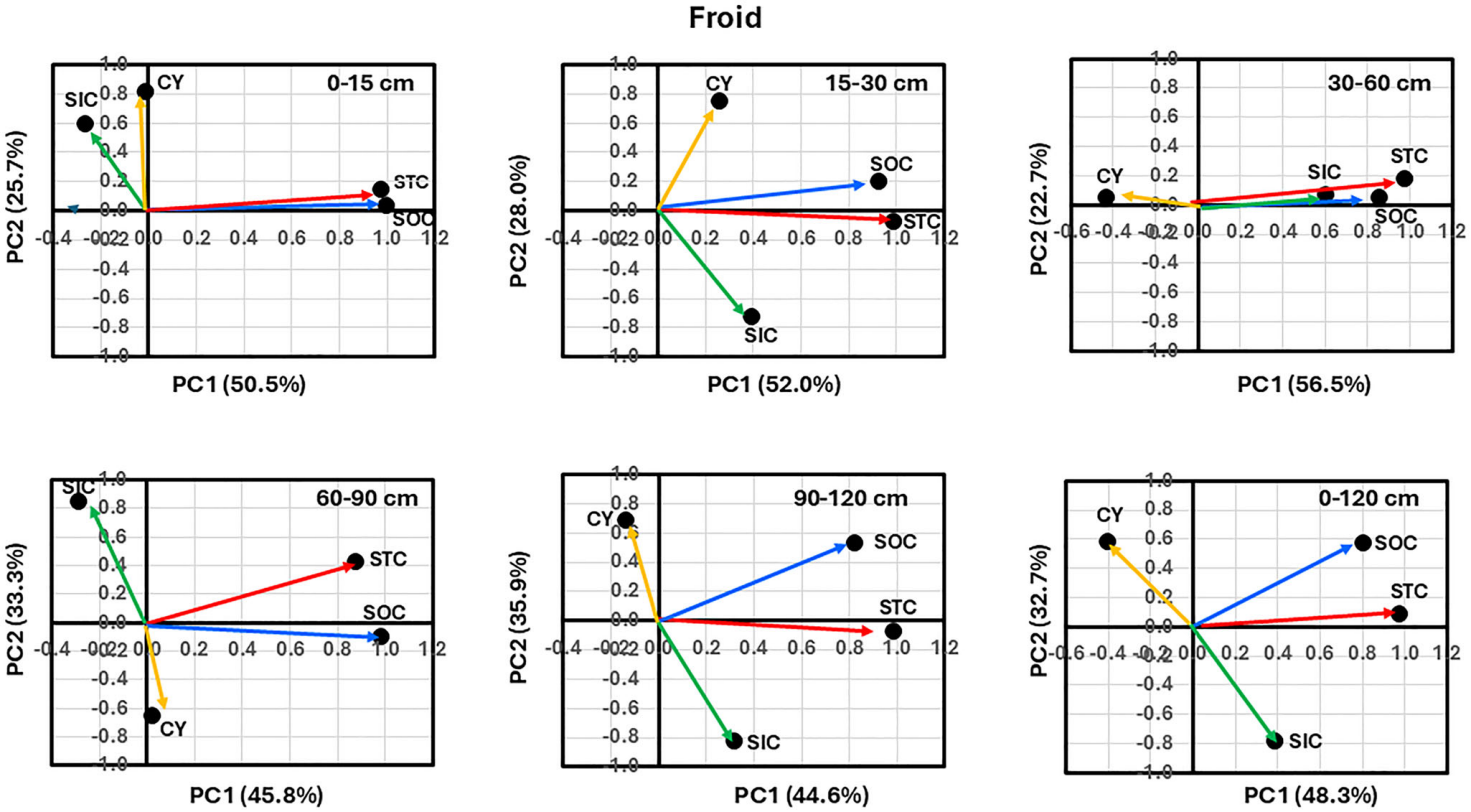
Principal component analysis for the relationships among soil C stocks at 0‐ to 15‐cm, 15‐ to 30‐cm, 30‐ to 60‐cm, 60‐ to 90‐cm, 90‐ to 120‐cm, and 0‐ to 120‐cm depths and crop yield for the crop rotation study at Froid. CY, crop yield; PC1, principal component 1; PC2, principal component 2; SIC, soil inorganic C; SOC, soil organic C; and STC, soil total C.

**FIGURE 2:**
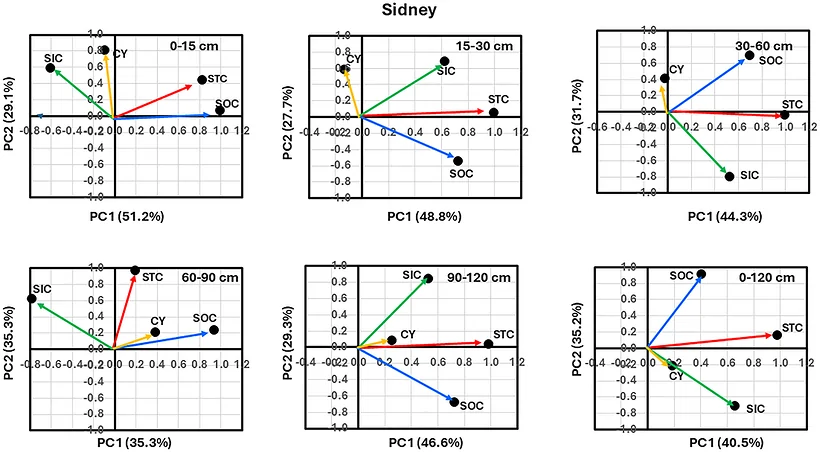
Principal component analysis for the relationships among soil C stocks at 0‐ to 15‐cm, 15‐ to 30‐cm, nd 30‐ to 60‐ to 90‐­ cm, 90‐ to 120‐­ m, and ‐0 to 120‐­ m  depths and crop yield for crop rotation studies at Froid. CY, crop yield; PC1, principal component 1; PC2, principal component 2; SIC, soil inorganic C; SOC, soil organic C; and STC, soil total C.

At Sidney, soil C stocks contributed from 35% of the total variability at 60–90 cm to 51% at 0–15 cm for PC1 for the relationship between soil C stock and crop yield (Figures 2 and 3). For PC2, soil C stocks contributed from 25% of the total variability at 0–60 cm to 35% at 60–90 and 0–120 cm. The STC contributed most of the variance for PC1 at all depths, except at 0–15 cm where both STC and SOC contributed the variance and at 60–90 cm where SOC contributed the most variance. The largest variance contribution at all depths in PC2 was crop yield, except at 60–90 cm where STC contributed the most variance. As at Froid, STC and SOC were positively related to each other at all depths, except at 60–90 cm where there was no relationship. The SIC was positively related to STC at all depths, except at 0–15 cm where no relationship occurred. The SIC did not relate to SOC at all depths. The relationship between soil C stocks at various depths and crop yield was variable. The STC at 0–15, 90–120, and 0–120 cm was positively related to crop yield. Similarly, SOC at 30–60, 60–90, 90–120, 0–30, 0–60, and 0–90 cm was positively related to crop yield. The SIC at 0–15, 90–120, and 0–120 cm was positively related to crop yield.

**FIGURE 3:**
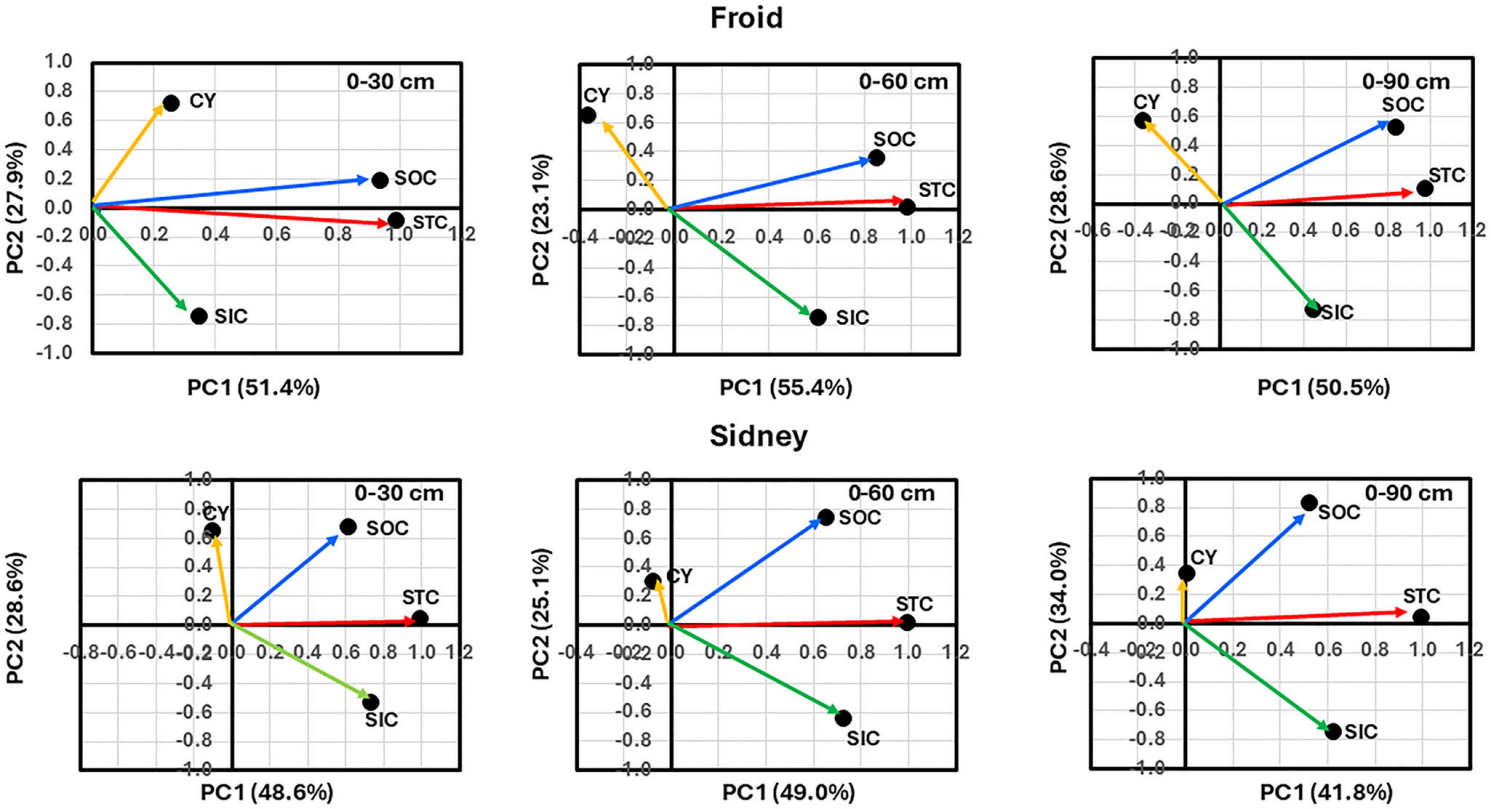
Principal component analysis for the relationships among soil C stocks at 0‐ to 30‐cm, 0‐ to 600‐cm, and 0‐ to 90‐cm depths and crop yield for the crop rotation study at Froid and Sidney. CY, crop yield; PC1, principal component 1; PC2, principal component 2; SIC, soil inorganic C; SOC, soil organic C; and STC, soil total C.

## 4 DISCUSSION

### 4.1 Nonlegume monocropping and diversified crop rotations increase soil carbon stocks

The greater crop residue C returned to the soil and higher residue C/N ratio may have increased STC and SOC at 0–15 cm for D compared to DNP, DP, DPC, DPN, and DPF at Froid (Table 4). Mean crop residue yield across years was greater for D and DPF than DC, DN, DP, DPC, and DF (Table 6). Sainju et al. (2025), from the same experiment, showed that STC was greater for D than other crop rotations due to increased crop residue C input and higher C/N ratio. The STC and SOC stocks are usually proportional to the amount of crop residue returned to the soil, as crop residue supplies C (Chahal et al., 2025; Halvorson & Schlegel, 2012; King et al., 2020; Plaza‐Bonilla et al., 2016; Rigon & Calonego, 2020; Zuber et al., 2018). Slower decomposition of the crop residue due to higher C/N ratio also increases SOC (Chahal et al., 2025; King et al., 2020; Rigon & Calonego, 2020; Zuber et al., 2018). The fact that crop rotation influenced STC and SOC only at 0–15 cm, but not at other depth layers, was probably due to the return of aboveground crop residue C at the soil surface, which also received belowground (root) residue C input compared to subsoil layers, which received C input only from the belowground residue (Chahal et al., 2025; King et al., 2020; Rigon & Calonego, 2020).

The greater STC at 15–30 cm for B and PWCS than other crop rotations and greater SOC at 0–15 cm for B, PBNS, PWCS, PWNS, and S than other crop rotations, except for PBCS and WP, at Sidney (Table 5) also indicate that increased aboveground crop residue C input and higher residue C/N ratio increased STC and SOC for continuous nonlegumes and highly diversified crop rotations compared to less diversified rotations, such as BP and SP. Mean crop residue yield across years was greater for PBNS, PWCS, and PWNS than other crop rotations (Table 6). This also could be the reason for greater SOC at 30–60 cm for PBCS than BP and SP, greater SOC at 90–120 cm for PWNS and W than BP, PBNS, S, and WP, and greater SOC at 0–120 cm for B, PBCS, PWCS, PWNS, and W than BP. It is likely that increased aboveground crop residue production also increased the yield of belowground residue, thereby increasing SOC at subsurface layers for continuous nonlegumes and diversified crop rotations.

The SIC at all depths was unaffected by crop rotation at Froid (Table 4). However, greater SIC at 90–120 cm for BP than other crop rotations, except B and S, and greater at 0–120 cm for B than other crop rotations, except BP and S, at Sidney (Table 5) were probably due to either reduced uptake of Ca and Mg by crops from lower crop yield or to translocation of SIC from the surface to subsurface layers due to the application of urea fertilizer at the soil surface. The H‐ions produced from NH_4_‐based N fertilizers can dissolve SIC at the surface soil and translocate into subsurface layers (Mikhailova & Post, 2006).

The greater significance of SIC and SOC at subsurface layers due to crop rotations at Sidney than at Froid was probably due to the variations in soil textures, as climatic conditions were almost similar at both sites. As Sidney (325 g kg^−1^) had greater clay concentration than Froid (170 g kg^−1^), it is likely that highly diversified crop rotations increased soil C stocks compared to less diversified rotations under fine soil texture at Sidney compared to under coarse soil texture at Froid. A possible mechanism for this could be the reduced decomposition of crop residue due to lower microbial activity in the fine‐textured soil, leading to increased accrual of SOC compared to rapid decomposition of the residue in the coarse‐textured soil. Changes in soil bulk density due to crop rotation probably had little effect on STC, SIC, and SOC stocks because bulk density was not affected by crop rotation at any depth, both at Froid and Sidney (Table 2). Because our studies were conducted in no‐till conditions that did not disturb soils, soil C stocks were not corrected for equivalent soil mass (Ellert & Bettany, 1995).

### 4.2 Diversified crop rotations increase crop yields

Increased water‐ and N‐use efficiencies and lower infections of weeds and pests may have enhanced crop yield for DCP compared to other crop rotations, except DP, at Froid, and enhanced for BP compared to other crop rotations, except SP and W, at Sidney (Table 6). Many researchers (Allen et al., 2014; Gan et al., 2009; Kirkegaard et al., 1999; Lenssen et al., 2014, 2020; Miller et al., 2002) have reported that crop rotation increased crop yield due to efficient use of water and N and reduced weed and pest pressure compared to continuous monocropping. Legume residues supply more N to succeeding crops due to biological N fixation than nonlegume residues, and legumes also use less soil water than nonlegumes, thereby increasing more soil water available to succeeding crops and enhancing their yields (Lenssen et al., 2014, 2020; Miller et al., 2002).

### 4.3 Soil carbon stocks relate variably to crop yields

The presence of lower SIC probably enhanced the relationship between SOC and STC at 0–15, 15–30, 30–60, 0–30, and 0–60 cm at Froid (Figures 1 and 2). However, the relationship decreased at 60–90, 90–120, 0–90, and 0–120 cm, probably due to the presence of greater SIC at subsurface layers. No wonder, SIC was not related to STC or SOC at all depth layers, except at 30–60 cm. At Sidney, SOC and STC at all depths were also related to each other (Figures 2 and 3), but the relationship was poorer than at Froid. This was probably due to greater SIC at all depths at Sidney than at Froid (Tables 1 and 3), which may have prevented the close relationship between STC and SOC at Sidney. Because SIC at all depths was not significantly affected by crop rotation in Froid and Sidney, except at 90–120 cm in Sidney (Tables 4 and 5), it may be obvious that the presence of carbonates may have prevented the close relationship between STC and SOC at deep soil layers rather than the effect of crop rotation on the relationship. These results were also supported by correlations values among STC, SIC, and SOC at various depths (Tables 7 and 8). Sainju et al. (2021) reported that STC and SOC at 0–15 cm were closely related to each other, but SIC was either negatively related to SOC or not related to STC and SOC, regardless of soil texture.

The non‐relationships between STC or SOC at all depths, except at 30–60 cm, and crop yield or the negative relationship at 30–60 cm at Froid (Figures 1 and 2) suggest that crops are not receiving benefits from increase in soil C stocks in coarse‐textured soils. It is likely that water‐holding capacity or availability of nutrients from the mineralization of organic matter is lower in coarse‐textured soil, thereby providing little or no benefits for crops (Diaz‐Zorita et al., 1999; Lal, 2020). This may also be the case for non‐relationships between STC or SOC at 0–15 and 15–30 cm and crop yield at Sidney (Figures 2 and 3). However, the positive relationships between SOC or STC at 60–90, 90–120, 0–30, 0–60, 0–90, and 0–120 cm and crop yield at this site suggest that fine‐textured soil at subsoil layers probably holds more soil water, which may have enhanced crop yields. Crop roots grow deeper into the soil profile to absorb water in dryland cropping systems (Cutforth et al., 2013). The greater relationship between SOC than STC at 30–60, 60–90, 0–30, 0–60, and 0–90 cm and crop yield at Sidney indicates that SOC may be a more reliable indicator for predicting crop yield than STC, especially in fine‐textured soil. This, however, contradicts the findings stated by Sainju et al. (2021, 2022), who reported that STC at 0–15 cm correlated more with crop yields than SOC in dryland cropping systems. Variations in SIC at 0–15 cm, which can be negligible or non‐existent at the surface soil, may have resulted in different relationships for STC or SOC at this depth with crop yields in this study compared to those reported by Sainju et al. (2021, 2022). It is difficult to obtain a close relationship between SOC and crop yield because yields are affected by multiple factors, such as climate, topography, and soil texture that confound the results (Lucas & Weil, 2012; van Es & Karlen, 2019). Correlation values among soil C stocks and crop yields (Tables 7 and 8) also supported the relationships between these parameters obtained by the PCA analysis.

The close relationship between SIC at 0–15 cm and crop yield at Froid and between SIC at 0–15, 15–30, 90–120, and 0–120 cm and yield at Sidney suggests that SIC can also increase crop yields by supplying basic cations, such as Ca and Mg. However, the negative relationship between SIC at other depths and crop yield at Froid and Sidney indicates that greater soil pH due to higher SIC may reduce nutrient availability and decrease crop yield.

### 4.4 Implications of the study

The results imply that highly diversified crop rotations increased both STC and SOC as well as crop yields compared to less diversified rotations. Continuous nonlegume monocropping also increased STC and SOC but reduced crop yields. Results were pronounced more for SOC than STC at subsurface compared to surface soil layers, especially for fine‐textured soil at Sidney. Soil C stocks were related variably with crop yields, but SOC was related more positively with crop yields than STC at subsurface compared to surface soil layers in fine‐textured soils. Therefore, SOC can be a preferable indicator for relating with crop yield compared to STC in dryland cropping systems. Enhanced SOC using diversified crop rotations can improve soil health by increasing soil physical, chemical, and microbial properties. Although SIC at some depth layers was related with crop yield, the nonsignificant effect of crop rotation for this parameter and the variable relationship with crop yield show that the parameter is less desirable to relate with crop yield.

## 5 CONCLUSIONS

Results from this study showed that highly diversified crop rotations and continuous nonlegumes increased STC and SOC stocks but not SIC stock compared to less diversified rotations at Froid and Sidney. Crop rotations also increased crop yields compared to continuous nonlegumes at both sites. The STC and SOC at all depths were related to each other, but did not relate to SIC. The SOC and STC at subsurface layers were related to crop yield at Sidney, but SIC at surface and subsurface layers were related variably with crop yield. The SOC was more related to crop yield than STC, especially in fine‐textured soil. The variable relationships among STC, SOC, SIC, and crop yields suggest that long‐term research >10 years may be needed to observe close relationship between soil C stocks and crop yields under dryland cropping systems in the US northern Great Plains because crop yields can be affected by soil and climatic conditions. Results suggest that SOC can be used to relate with crop yields better than STC and SIC in dryland cropping systems.

## AUTHOR CONTRIBUTIONS

**Upendra M. Sainju**: Conceptualization; data curation; formal analysis; investigation; methodology; writing—original draft; writing—review and editing. **Brett L. Allen**: Data curation; investigation; project administration; resources; supervision; writing—review and editing. **Jalal D. Jabro**: Data curation; investigation; validation; visualization; writing—review and editing. **William B. Stevens**: Data curation; project administration; resources; visualization; writing—review and editing.

## CONFLICT OF INTEREST STATEMENT

The authors declare no conflicts of interest.
